# Sinonasal Manifestations in Cystic Fibrosis

**DOI:** 10.1155/2012/789572

**Published:** 2012-08-05

**Authors:** Karin P. Q. Oomen, Max M. April

**Affiliations:** Divison of Pediatric Otolaryngology-Head and Neck Surgery, Department of Otolaryngology-Head and Neck Surgery, Weill Cornell Medical College, 428 East 72nd Street, Suite 100, New York, NY 10021, USA

## Abstract

Cystic fibrosis is a genetic disease, characterized by accumulation of thickened mucous secretions in exocrine glands. Although the major clinical manifestations of the disease are pancreatic and pulmonary disease, the majority of cystic fibrosis patients will develop sinonasal manifestations as well. This paper outlines the etiology, evaluation, and management of the nasal and sinus manifestations in patients with cystic fibrosis.

## 1. Introduction

Cystic fibrosis (CF) is an autosomal recessively inherited disease, caused by mutations in the CF gene located at chromosome 7. The CF gene encodes the CF transmembrane regulator (CFTR), a membrane bound protein capable of chloride ion transport [1]. A deficiency of the CFTR leads to chloride channel dysfunction at the epithelial cells lining the airway and exocrine glands, causing accumulation of thickened mucous secretions.

Although the dominant clinical features of CF are lower respiratory tract infections and pancreatic insufficiency, the vast majority of CF patients will also develop chronic rhinosinusitis due to sinonasal mucus accumulation [2–5]. Because of the considerable morbidity associated with sinus disease and the growing belief that sinonasal involvement may worsen pulmonary manifestations, the otolaryngologist has become increasingly involved in evaluation and management of CF patients.

This paper will provide an aid in clinical decision making by outlining the etiology, evaluation, and management of the nasal and sinus manifestations in patients with CF from an evidence based perspective.

## 2. Etiology and Evaluation

Rhinosinusitis is an inflammatory process of multifactorial etiology, involving the mucosa of the nose and one or more sinus. Factors contributing to the pathology of rhinosinusitis are mucociliary impairment, infection, allergy, mucosal edema, and, rarely, physical obstructions caused by morphological or anatomical variation in the nasal cavity or paranasal sinus [6]. The prevalence of rhinosinusitis in the CF population borders on 100%, according to a combination of symptoms, physical and radiologic findings [3–5]. The high susceptibility for sinus disease in CF patients may be related to altered properties of their mucous secretions, leading to impaired mucociliary clearance. Obstruction of the ostia leads to ciliary injury, mucosal edema, and general inflammation, which is further exacerbated by chronic colonization, in most cases with pathogens of the upper and lower respiratory tract such as *Pseudomonas* species, *Staphylococcus aureus*, and nontypable *Haemophilus influenzae* [7]. Furthermore, genetic studies have suggested that the CFTR mutation responsible for CF might in itself be a predisposing factor for sinus disease, by demonstrating an increased occurrence of CFTR mutations in the general population with chronic rhinosinusitis [8, 9].

The most frequent presenting symptoms of sinus disease in CF are nasal congestion and purulent nasal discharge, but headache, mouth breathing, anosmia, and hyposmia related to chronic sinus disease are commonly reported as well. Physical findings may vary, but are mostly due to purulent drainage and mucosal changes resulting in nasal obstruction. Nasal polyps may be identified on anterior rhinoscopy or nasal endoscopy in up to 86% of children with CF, but the frequency varies in different populations and study groups [10–16]. Other findings include swollen turbinates and lymphoid hyperplasia in the posterior pharynx. 

**Figure 1:**
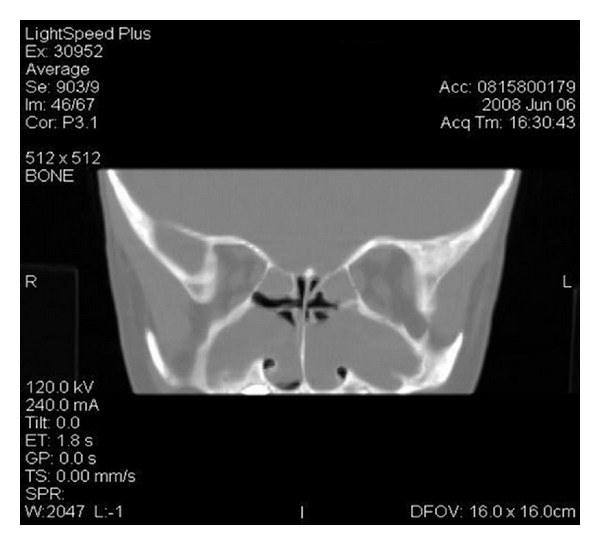
Coronal CT image of bilateral medial displacement of the lateral nasal wall in a pediatric CF patient.

Nearly all patients with CF show radiologic evidence of mucosal sinus disease, whether or not sinonasal symptoms are present [17]. To establish the relationship between CF and radiological sinus abnormalities more accurately, April et al. [18] studied the computed tomography (CT) scan findings of 58 CF patients. Bilateral medial displacement of the lateral nasal wall in the middle meatus and uncinate process demineralization were the most commonly encountered radiological findings, prevalent in 74% of patients. (Figure 1) Previous studies have shown that the extent of sinus disease on CT images does not correlate with symptom severity in patient with chronic rhinosinusitis [15, 19]. However, given the rate of anatomical variations and abnormalities of the sinus in CF, the CT scan remains of great value in the preoperative work-up of these patients, irrespective of its diagnostic role. 

## 3. Management

### 3.1. Medical Management

 As survival continues to improve for CF patients, management of sinonasal manifestations has become increasingly important. Medical management of sinusitis in CF generally consists of local and/or systemic anti-inflammatory medications and antibiotics. Hadfield et al. [20] have shown that local anti-inflammatory agents, such as nasal steroids, may reduce polyp size in patients with CF, although they did not demonstrate a significant reduction in associated sinus symptoms.

Systemic glucocorticoids are commonly used in CF patients to reduce pulmonary symptoms, and might have a beneficial effect on sinonasal manifestations as well. Unfortunately, no evidence exists on the effects of oral steroids on sinusitis in CF patients. A recent Cochrane review of randomized controlled trials of oral corticosteroids in CF patients demonstrates slowing of progression of pulmonary disease, reduction of hospitalization for respiratory exacerbations, and improved quality of life, but does not directly address the effect on sinonasal symptoms [21]. The positive effect of oral glucocorticoids on chronic sinusitis in the general population [22, 23] suggests a role for these agents in management of sinus disease in CF patients as well. 

Recently, a randomized controlled trial by Ramsey et al. [24] demonstrated substantial improvements in lung function, rate of pulmonary exacerbations, and patient-reported respiratory symptoms associated with the use of a new therapeutic agent, the CFTR potentiator Ivacaftor, in a subset of CF patients. These findings might represent an important milestone in the development of treatments addressing the underlying cause of CF. Effects on sinonasal manifestations, however, were not measured.

Antibiotic therapy in CF is generally directed toward CF related organisms that occur in both the upper and lower airway. Both sputum and middle meatus cultures are used as a guide for antibiotical therapy, and previous studies have shown the most commonly cultured pathogens to be *Pseudomonas aeruginosa* and *Staphylococcus aureus* [25, 26]. CF patients generally use oral antibiotics such as ciprofloxacin or azithromycin as a prophylaxis to prevent infections of the lower and upper airway or to control present infections [27]. Inhaled therapy with antibiotics such as tobramycin, colistin, and aztreonam is often used as well in order to improve lung function by aiming at colonized bacteria, but their efficacy in treating upper airway manifestations remains unknown [28–30]. Unfortunately, aminoglycosides such as tobramycin are known to cause sensorineural hearing loss and damage to the labyrinth with long-term use [31], which is in itself another reason for the otolaryngologist to be involved in CF patient care. The efficacy of oral antibiotics in treatment of chronic rhinosinusitis in otherwise healthy patients [22] suggests a role for antibiotics in the CF population with sinus disease as well, and a previous study has demonstrated that long-term systemic treatment with macrolides may reduce nasal polyp size in patients with CF [32]. 

### 3.2. Surgery

Unfortunately, many CF patients will fail medical management of sinusitis, and approximately 20–25% of patients ultimately undergo sinus surgery [33]. As the survival of patients with CF increases, the number of CF patients requiring sinus surgery will likely grow. Sinus surgery is not only recommended because of medically intractable symptomatic nasal polyposis and trapped secretions in the sinus, but also as a preventive and preparatory measure for lung transplant candidates. After lung transplantation, a major cause of morbidity and mortality is *Pseudomonas aeruginosa* pneumonia, which is thought to originate from sinus colonization [2]. 

As in the general population, traditional techniques for sinus surgery in CF have included simple polypectomy and open ethmoidectomy or Caldwell-Luc procedures. Attempts at treatment of sinusitis in CF patients in the past with polypectomy alone have resulted in initial symptom relief but recurrence rates of more than 80% [34–36]. More extensive surgery such as ethmoidectomy or Caldwell-Luc reduced recurrences to 45–60% with a 2 to 8 year follow-up [35, 36]. The introduction of more advanced sinus surgery techniques in recent years has fuelled hopes for successful and less invasive surgical management of sinusitis in CF. Endoscopic sinus surgery (ESS) has been shown to be safe and effective for the treatment of sinus disease in patients with CF in many studies [11, 37–43]. Some studies have shown a significant improvement in both sinonasal symptoms and quality of life following ESS, [30, 42, 43] whereas others have shown a reduced need for revision surgery following ESS as compared to conventional surgery [44]. Outcomes of aggressive sinus surgery might be less favorable in CF patients. A recent study by Georgalas et al. [45] on long-term results of Draf type III (modified endoscopic Lothrop) frontal sinus drainage in a cohort of 122 patients demonstrated the highest risk of restenosis of the frontal sinus ostium to be among CF patients.

Balloon catheter sinuplasty (BCS) was introduced as a new therapeutic modality of chronic rhinosinusitis in otherwise healthy adults around 2006, and has been shown to be equally effective as ESS [46]. More recently, BCS has been evaluated for safety and efficacy of treatment of chronic rhinosinusitis in the pediatric population. BCS appears to be safe and effective, according to recent cohort studies, [47–50] and in contrast to ESS, it does not involve tissue removal and spares mucosa. This noninvasive technique may be particularly beneficial in the pediatric age group and thus in a group of CF patients. None of the studies regarding BCS have focused on CF patients yet, but its safety and efficacy in the pediatric population might suggest a role for BCS in the treatment armamentarium of CF.

Of the CF patients undergoing ESS, a high proportion will undergo revision surgery [51]. This is explained by the fact that CF is a chronic disease, and impairment of mucociliary transport remains the underlying problem through which disease may recur even after initially successful surgery. Previous studies have focused on tools to predict future outcomes after sinus surgery in CF patients, in order to provide an aid in decision making for physicians. A previous prospective review demonstrated a significantly increased likelihood of undergoing revision surgery in patients with predominantly nasal blockage symptoms, as compared to those with predominantly infective sinonasal symptoms such as mucopurulent rhinorrhea and pain [52]. This study did not show a significant association of extent of disease on initial CT scan with rate of revision surgery in CF patients. Conversely, a retrospective review by Becker et al. [51] demonstrated that CF patients with higher degrees of sinus opacification—expressed in higher Lund-Mackay scores—at their initial CT scan were significantly more likely to undergo repeat sinus surgeries. A recent retrospective study by Rickert et al. [53] concluded that preoperative grading of nasal polyposis in patients with CF may provide a helpful tool in assessing the future likelihood of revision ESS, by demonstrating a positive correlation between nasal polyposis grade and rate of revision surgery. The high rates of revision surgery raise the question whether CF patients gain an actual long-term benefit from ESS. Several studies have shown initial positive symptom outcomes after sinus surgery in CF, but a 50 to 100% recurrence rate in a follow-up period of approximately 2 years [37, 40, 41, 52, 54]. A large proportion of studies, however, measure their postoperative outcome largely on the basis of CT images, which are known to correlate less well with symptom severity. 

Some authors have argued whether sinus surgery has a positive effect on the pulmonary status of CF patients. An effect of ESS on pulmonary status was initially reported in a small study among adult CF patients, which showed that pulmonary symptoms, but not function, improved after sinus surgery [55]. Madonna et al. studied the effects of sinus surgery on pulmonary function in CF and did not demonstrate a significant improvement [56]. On the other hand, a larger retrospective study showed that although CF patients did not have a significant difference in pulmonary function test results or steroid requirements, they did have a reduced need for hospitalization in the 6 months following ESS [57]. Specific effects of ESS on rhinosinusal manifestations were not reported in this study. The most recent study on this subject, a retrospective medical record review of young pre-lung transplant CF patients by Osborn et al. [58], showed no improvement of pulmonary function tests and no alteration of respiratory tract microbial pathogen patterns following ESS.

Due to underlying issues such as acquired coagulopathies and advanced pulmonary disease, perioperative morbidity has been assumed to be higher in CF patients. However, a previous study by Albritton and Kingdom demonstrated a low complication rate for ESS in this patient group, compared to non-CF ESS complication rates reported in the literature [38]. Another previous study did not show association of ESS or open sinus surgery with increased rates of pulmonary complications or postoperative bleeding in CF patients [39]. 

Postoperative management for ESS in CF patients may differ from postoperative management in the general population because of the higher recurrence risk of sinus disease with CF. One study has shown that postoperative serial antibiotic lavage of the sinus may reduce recurrence requiring surgery for a period of at least 2 years in CF patients [44]. However, a study by Rickert et al. demonstrated a very low (<30%) overall rate of revision surgery in CF patients without the use of antibiotic lavage, when compared to other studies [53].

## 4. Future Directions

Although a large body of the literature addresses rhinosinusal manifestations in CF, certain gaps in evidence on related issues remain.

The exact role of the CT scan in the diagnostic work-up of patients with sinonasal manifestations of CF has remained subject to debate. Although the definition of rhinosinusitis may be based on symptoms and signs only, [59] many clinicians use CT scans to help define its actual presence. Previous studies have shown that the extent of sinus disease on CT images does not correlate with symptom severity in patients with chronic rhinosinusitis, both in the CF and the otherwise healthy population [15, 16, 19]. Studies on CT scan findings of the sinus in CF patients have established that specific abnormalities, such as bilateral uncinate process demineralization and medial displacement of the lateral nasal wall, might be CF defining, and should raise suspicion of CF in any patient with sinonasal symptoms, especially children [17, 18]. These findings suggest a diagnostic role of the CT scan in sinonasal manifestations of CF. 

To the same extent, it remains uncertain whether culturing is an important diagnostic tool in sinus disease associated with CF. Both sputum and middle meatus cultures are currently used as a guide for antibiotical therapy, and previous studies have shown the most commonly cultured pathogens to be *Pseudomonas aeruginosa* and *Staphylococcus aureus* [25, 26]. However, further studies are required to comprehend the role of specific microorganisms in the pathogenesis of sinus disease in CF and the association of culture results with clinical severity. 

Medical treatment for sinonasal manifestations in CF needs to be further studied to establish the role of both systemic antibiotics and anit-inflammatory agents such as glucocorticoids. Antibiotical therapy may need to be studied specifically for its potentiality of inflicting sensorineural hearing loss, and future treatment protocols might incorporate annual audiometry of CF patients on the basis of such studies.

 When it comes to the effectiveness of ESS for sinus disease in CF, long-term results are few. There remains a need for prospective trials on the effect of sinus surgery in patients with CF. Outcome measures should not only focus on radiological appearance of the sinus and sinonasal symptoms, but also on quality of life and need for hospitalizations for upper and lower respiratory tract problems. Future research might also focus on new surgical techniques to treat sinonasal manifestations. The safety and efficacy of BCS in treatment of chronic rhinosinusitis in the otherwise healthy pediatric population suggest a potential role for BCS in the treatment of sinonasal manifestations of CF. 

The increasing life expectancy of CF patients might raise the question if pediatric and adult CF populations require different treatment approaches with respect to sinonasal manifestations. Most studies on effects of ESS in CF have been carried out in mixed adult and pediatric populations. In order to differentiate between the effect of ESS on sinus disease in the pediatric and adult population, separate studies for each age group might be needed. Such studies might hold the promise for specific age appropriate surgical treatment of CF patients.

Given the high recurrence rate of sinus disease in this population, studies on perioperative management of CF patients undergoing sinus surgery are also needed, with a focus on pre and postoperative antibiotics and anti-inflammatory agents. Such studies might provide an aid in prevention of disease recurrence and revision procedures. The lack of effect of ESS on pulmonary function test results in previous retrospective studies [55, 56, 58] highlights the need for prospective assessment of pulmonary effects, as well as postoperative quality of life improvement and of adjunct medical therapy efficacy for pulmonary symptoms.

## 5. Summary

Sinonasal manifestations are prevalent in almost all patients with CF, whether represented by signs, symptoms, or radiologic findings (grade B, D evidence) [3–5]. Nasal polyps are the most frequently encountered findings on physical examination and may occur in up to 86% of patients (grade C-D evidence) [10–16]. Virtually all CF patients demonstrate radiological evidence of sinus disease, but the extent of disease does not correlate with symptom severity (grade C-D evidence) [17, 59]. The most prevalent radiological abnormalities in CF have shown to be bilateral uncinate process demineralization and medial displacement of the lateral nasal wall (grade C evidence) [17, 18].

Medical treatment of sinus disease in CF may consist of local and/or inflammatory agents and antibiotics. However, many patients will ultimately fail medical therapy and undergo sinus surgery (grade B evidence) [33]. ESS has been proven to be a safe and effective treatment of sinus disease in CF (grade B-C evididence). [11, 37–43] Unfortunately, a high proportion of CF patients will eventually require revision surgery because of recurrence or persistence of sinus disease (grade C evidence) [51]. The identification of factors associated with an increased likelihood of need for revision sinus surgery has been aimed at by many studies, with equivocal outcomes (grade B-C evidence) [51–53]. Studies on the effect of sinus surgery on pulmonary function have shown improvement of pulmonary symptoms and a reduced need for hositalization, but no differences in pulmonary function test outcomes (grade C evidence) [55–58]. No association has been found between sinus surgery and increased rates of postoperative complications, including hemorrhage (grade C evidence) [38, 39]. Surgical revision rates in patients with nasal polyps are much higher than in patients without polyps (grade C evidence) [53]. Evidence grades and conclusions are summarized in Table 1.

## Figures and Tables

**Table 1:** Conclusions and grades of evidence.

Category	References	Description	Level of evidence	Evidence grade	Conclusion
Sinonasal manifestations in CF	[3, 4] [5]	Expert opinion Individual cohort studies	5 2b	D B	Sinonasal manifestations are prevalent in almost all CF patients

Presence of nasal polyps in CF	[10–14, 16] [15]	Expert opinion Lower quality cohort study	5 3b	D C	Nasal polyps may occur in up to 86% of CF patients

Radiological evidence of sinus disease in CF	[17] [59]	Nonconsecutive cohort study Expert opinion	3b 5	C D	Virtually all CF patients demonstrate radiological evidence of sinus disease

Radiological abnormalities	[17, 18]	Retrospective chart review	4	C	The most prevalent radiological abnormalities in CF are bilateral uncinate process demineralization and medial displacement of the lateral nasal wall

Failing of medical therapy	[33]	Retrospective cohort study	2b	B	Many CF patients will ultimately fail medical therapy and undergo sinus surgery

ESS as a treatment for sinus disease in CF	[11, 37–43]	Retrospective cohort studies, individual case control studies and retrospective chart reviews	2b, 3, 4	B-C	ESS is a safe and effective treatment of sinus disease in CF

Revision sinus surgery	[51]	Retrospective cohort study	2b	B	A high proportion of CF patients will eventually require revision sinus surgery

Risk factors for revision surgery	[51]	Retrospective cohort study	2b	B	The identification of factors associated with an increased likelihood of need for revision sinus surgery has resulted in equivocal outcomes
	[52, 53]	Lower quality cohort study	4	C	

Effects of surgery on pulmonary outcomes	[55–58]	Lower quality cohort studies	4	C	There is an improvement of pulmonary symptoms and a reduced need for hospitalizations after sinus surgery in CF, but no differences in pulmonary function test outcomes

Postoperative complications	[38, 39]	Lower quality cohort studies	4	C	There is no association between sinus surgery in CF and increased rates of postoperative complications

Surgical revision rates	[53]	Lower quality cohort study	4	C	Surgical revision rates CF patients with polyps are much higher than in those without polyps
